# Supplementary material for: Consistent predator-prey biomass scaling in complex food webs
Source: Nat Commun. 2022 Aug 25;13:4990. doi: 10.1038/s41467-022-32578-5 (PMC9411528; doi:10.1038/s41467-022-32578-5)
Supplement: Supplementary file 1 — Supplementary Information [file 41467_2022_32578_MOESM1_ESM.pdf]

Consistent predator-prey biomass scaling in complex food webs

Daniel M. Perkins, Ian Hatton, Benoit Gauzens, Andrew D. Barnes, David Ott, Benjamin Rosenbaum, Catarina Vinagre & Ulrich Brose

Contents:

Appendix S1 & S2

Tables S1 to S5

Figures S1 to S4

Appendix S1 | Linking predator-prey biomass scaling relations within and across food webs

Adopting a food web approach permits testing whether predator-prey scaling across food webs (Fig. 1d), can be extrapolated from scaling relations within food webs (Fig. 1c) - and *vice versa* – providing a basis to link biomass distributions across levels of biological organization. We would expect that within-web scaling (between predator populations and their prey) implies similar across-web (ecosystem-level predator-prey) scaling in the limit of very simple systems, but that predictions are more involved in more complex food webs with variation in size structure, prey partitioning among predators and degree of omnivory.

In a simple, but not very realistic case, we consider the case of no omnivory, and no systematic change in size structure, where one predator population feeds exclusively on one prey population along a gradient. In this case, no matter how we aggregate prey is likewise how we aggregate predators, and so any aggregate predator-prey scaling will approach the same exponent as the largest within-web predator-prey exponent.

Assuming that all predator-prey populations scale with a similar exponent k, we can write aggregate prey ecosystem biomass (B), consisting of populations i, as $B = \sum_i B_i$. If size structure is invariant (e.g. a constant predator-prey body mass ratio), then the fraction of prey biomass in population i is independent of the total biomass density B: $f_i = B_i/B$.

It follows that population predator-prey scaling ($C_i = z_i B_i^k$, where C_i is predator and z_i is a constant coefficient for population i) implies community predator-prey scaling:

$$C = \sum_i C_i = \sum_i z_i B_i^k = \sum_i z_i (f_i B)^k = \left(\sum_i z_i (f_i)^k\right) B^k$$

The term in brackets on the right hand side should be a constant, so within-web scaling implies across-web scaling (at least in our highly simplified scenario).

It becomes more complicated when we consider that one predator population is sharing some or all prey populations with other predators. We have undertaken preliminary simulations

where predator species share two or three prey species with other predators. Although we find that aggregate across-web predator-prey scaling still maintains the same scaling as the within-web scaling, we hesitate to speculate further given the many dimensions in which food web properties might vary. These include variation in omnivory and size structure, which we know are highly variable and possibly systematic. Further theoretical work is needed to know if constancy, or lack of systematic variation, in these properties is sufficient to predict scaling across webs from the aggregate of within-web scaling.

Appendix S2 | Theoretical predictions for predator-prey biomass scaling

Many strands of ecological theory provide predictions for the distribution of biomass between predator-prey pairs and along food chains. However, we are not aware of any theory that makes predictions consistent with the sub-linear scaling of the predator-prey power law (Fig. 1a).

Predator-prey theory models the dynamics of feeding interactions, and has traditionally focused on two distinct trophic levels, rather than on networks of highly omnivorous food webs [1]. For a given model (Appendix Table 1), we relate the equilibrium predator and prey biomass across a simulated gradient of total community biomass, and ask whether it recovers the empirical scaling observed. A community biomass or 'enrichment' gradient can be modeled by varying a parameter in the prey growth term (Appendix Table 1), which may be exponential or logistic, or else in the predator-prey interaction term, which may be a linear or saturating functional response [1–3]. In Appendix Fig. 1 we show that equilibrium predictions for three classic models are not consistent with sub-linear predator-prey scaling, either because they are not stable [4,5] (a), do not provide meaningful relations [1,3] (b) or predict an exponent of 1 [6,7](c).

We can obtain consistent sub-linear biomass scaling by setting prey growth to have the same exponent as predator-prey biomass scaling [2,4–7] (Appendix Fig. 1d). Sub-exponential scaling of prey production with biomass (with an exponent of ~¾), stabilizes simple models such as Lotka-Volterra, and gives similar (~¾ power) equilibrium predator-prey biomass scaling. Two possible reasons explain sub-linear prey productivity scaling: (1) allometric constraints on individual rates which scale allometrically with body mass and could lead to sub-linear community scaling with a systematic relation of body size to biomass [8], and/or (2) density dependent effects - i.e. submaximal individual growth [2]. Hatton et al. [2] found that

mean prey body mass varied little over the prey biomass gradient and thus their results are consistent with the role of density dependent effects on population growth. Alternatively, we could introduce 'interference scaling' in the functional response or density dependence in the predator mortality (exponent ~1⅓). Despite the various possibilities, there is, as yet, no firm basis from classic predator-prey theory on which to understand sub-linear biomass scaling.

Appendix Table 1 | Predator-prey model equations used to generate the phase space isoclines in Appendix Fig. 1. In addition to the classic Lotka-Volterra model [4,5] (a), we include a typical top-down control model such as Rosenzweig-MacArthur [1,3] (b), and a bottom-up control model such as ratio-dependence [6,7] (c). Finally, we show a modified Lotka-Volterra model, where prey growth is sublinear ($k < 1$) [2]. In all equations, B is prey biomass and C is predator biomass. In addition, r is a prey growth constant (with different units in a-d); q is predator-prey interaction strength; g is predator growth conversion efficiency; h is sometimes referred to as predator handling time, controlling the saturation of the functional response; and m is predator mortality rate. A biomass gradient is modeled by varying q in (a) and (d), and by varying K in (b) and (c).

	Prey equation	Predator equation
a) Lotka-Volterra	$\frac{dB}{dt} = rB - qBC$	$\frac{dC}{dt} = gqBC - mC$
b) *Rosenzweig-MacArthur*	$\frac{dB}{dt} = rB\left(1 - \frac{B}{K}\right) - \frac{qBC}{1 + hB}$	$\frac{dC}{dt} = \frac{gqBC}{1 + hB} - mC$
c) *Ratio-dependence*	$\frac{dB}{dt} = rB\left(1 - \frac{B}{K}\right) - \frac{qBC}{C + hB}$	$\frac{dC}{dt} = \frac{gqBC}{C + hB} - mC$
d) *Sublinear prey growth*	$\frac{dB}{dt} = rB^k - qBC$	$\frac{dC}{dt} = gqBC - mC$

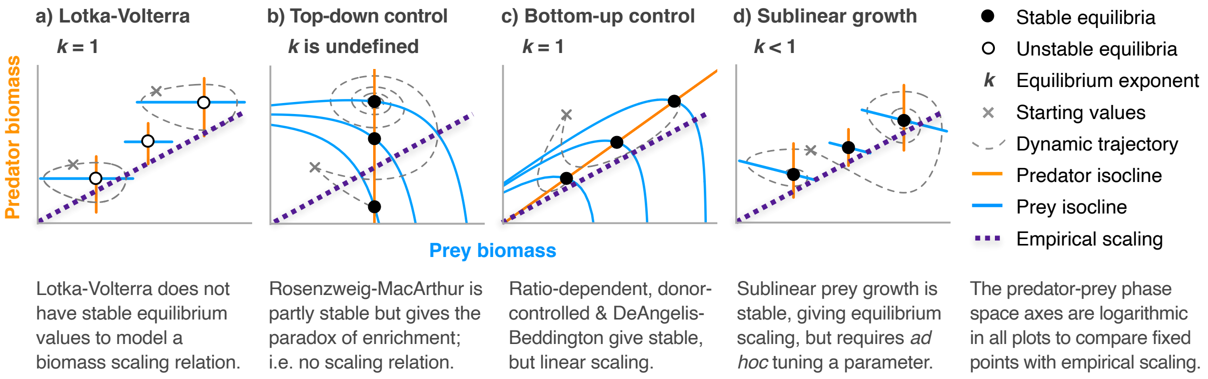

Appendix Fig. 1 | Simple predator-prey model predictions across simulated biomass gradients. Gradients are simulated by varying interaction strength in (a) and (d), and by varying the carrying capacity for prey logistic growth in (b) and (c). Models (a) to (c) do not make predictions consistent with sublinear predator-prey biomass scaling (predator-prey biomass scaling exponent, k, either equal to 1 or undefined). Model (d) makes realistic predictions ($k < 1$) but requires that prey growth is sub-exponential, which is an assumption that requires new theory. Predator-prey model equations used to generate the phase space are provided in Appendix Table 1.

Body size scaling theory is focused on explaining the origin of size-scaling, such as the ~¾ scaling of metabolism across classes of organisms [9,10] or linking different variables through their scaling exponents to make novel predictions [11,12]. Metabolic scaling theory would seem particularly relevant given the great variety of individual-level rates that scale with body size near ¾, including fundamental variables such as growth and metabolism [11,12]. However, these theories and empirical relations are focused at the individual level, and thus less relevant to aggregates of individuals in a community (but see [8,13,14]).

One exception is size-spectrum theory, which aims to explain the observation that, for whole ecosystems, biomass is approximately evenly distributed across logarithmic body size classes [15,16]. This pattern has been observed in primarily aquatic ecosystems, but also occasionally on land [17,18] and appears to hold across all ocean life from bacteria to whales [19]. Static scaling models of size spectra often assume that the predator-prey body mass ratio (PPmR), and trophic transfer efficiency (ratio of predator to prey production) do not vary

with total system biomass [13,16]. These measures indicate from which size class predators obtain their energy, and how efficiently that energy is utilized by any given predator to maintain its biomass [20].

Assuming both an even distribution of biomass across size classes, and a constant variation in prey body mass, however, suggests an unchanging trophic biomass pyramid (all else being equal), and is thus inconsistent with sub-linear predator-prey scaling. Such scaling means that the predator-prey biomass ratio (PPBR; not to be confused with PPmR) is declining with prey biomass (Appendix Fig. 2 a), and therefore the shape of the biomass pyramid (Appendix Fig. 2 b) becomes more bottom heavy with biomass (Appendix Fig. 2 e). To make size spectrum theory consistent with sub-linear biomass scaling, considering only the variation in prey body mass and the biomass spectrum slope (Appendix Fig. 2 c and d, respectively), at least one of these variables should vary.

If the prey base of any given predator is constant, then the slope of the biomass pyramid should become more negative (Appendix Fig. 2 f), paralleling changes in pyramid shape (Appendix Fig. 2 e). If instead the biomass spectrum slope is constant, then a higher biomass indicates predators should be obtaining prey from a larger set of size classes to maintain a more bottom-heavy biomass pyramid. This translates into a decline in variance of PPmR at higher biomass (Appendix Fig. 2 g). However, there is, as yet no evidence that size spectra slopes or PPmR vary systematically with total community biomass.

More likely than the possible variations in these simple static properties (Appendix Fig. 2), are changes in dynamical variables along a biomass gradient, such as flux rates, trophic transfer efficiencies and/or productivities with biomass.

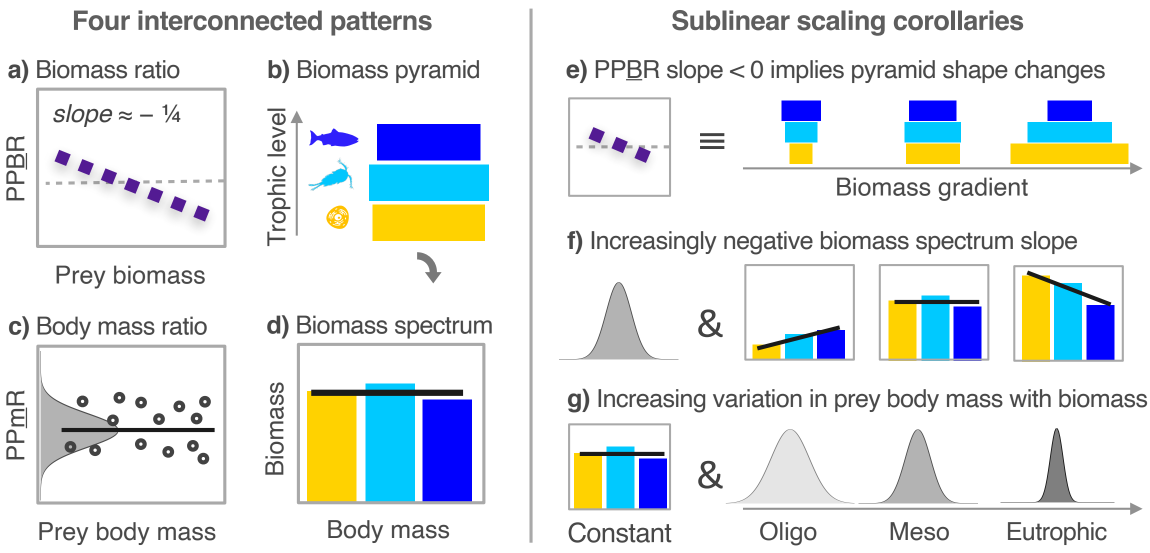

Appendix Fig. 2 | Predator-prey biomass scaling and the size-spectrum. Sub-linear predator-prey scaling gives a declining predator-prey biomass ratio (a) along a biomass gradient (PPBR slope < 0). This biomass scaling implies the trophic biomass pyramid (b) becomes more bottom-heavy with increasing biomass, as shown in (e). The biomass pyramid (b) can also be related to body size, since in aquatic systems, big eats small. Trophic level is thus broadly related to body mass through the predator-prey body mass ratio, PPmR (c). In (c), we consider the variation in prey body mass relative to two different predator masses, with the larger predator having a much broader prey base. To relate the variation in prey body mass for any given predator (c) to the biomass ratio, PPBR (a), we relate body mass to biomass through the biomass spectrum (d), which is the distribution of biomass across logarithmic body size classes, known to be approximately even. For PPBR to have slope < 0 (sublinear predator-prey scaling, as in a), there are at least two possible corollaries. If the variation in prey body mass is constant, then it implies that the slope of the biomass spectrum should become more negative, mirroring changes in pyramid shape (f). If on the other hand, the biomass spectrum slope is constant, then the variation in prey body mass for any given predator should increase with increasing biomass, translating into a smaller PPmR variation (g). This means that at high biomass, any given predator obtains prey from more size classes than at low biomass, and so the predator-prey biomass pyramid becomes more bottom-heavy. Axes are log in (a), (c) and (d).

1. McCann, K. S. *Food webs (MPB-50)*. (Princeton University Press, 2011).
2. Hatton, I. A. *et al.* The predator-prey power law: Biomass scaling across terrestrial and aquatic biomes. *Science.* **349**, aac6284 (2015).
3. Rosenzweig, M. L. Paradox of enrichment: destabilization of exploitation ecosystems in ecological time. *Science.* **171**, 385–387 (1971).
4. Lotka, A. J. *Elements of physical biology*. (Williams & Wilkins, 1925).
5. Volterra, V. Fluctuations in the abundance of a species considered mathematically. *Nature* **118**, 558–560 (1926).
6. Arditi, R. & Ginzburg, L. R. Coupling in predator-prey dynamics: ratio-dependence. *J. Theor. Biol.* **139**, 311–326 (1989).
7. Arditi, R. & Ginzburg, L. R. *How species interact: altering the standard view on trophic ecology*. (Oxford University Press, 2012).

8. Schramski, J. R., Dell, A. I., Grady, J. M., Sibly, R. M. & Brown, J. H. Metabolic theory predicts whole-ecosystem properties. *Proc. Natl. Acad. Sci.* **112**, 2617–2622 (2015).
9. West, G. B., Brown, J. H. & Enquist, B. J. A general model for the origin of allometric scaling laws in biology. *Science.* **276**, 122–126 (1997).
10. Banavar, J. R. *et al.* A general basis for quarter-power scaling in animals. *Proc. Natl. Acad. Sci.* **107**, 15816–15820 (2010).
11. Brown, J., Gillooly, J., Allen, A., Savage, V. & West, G. Toward a metabolic theory of ecology. *Ecology* **85**, 1771–1789 (2004).
12. Hatton, I. A., Dobson, A. P., Storch, D., Galbraith, E. D. & Loreau, M. Linking scaling laws across eukaryotes. *Proc. Natl. Acad. Sci.* **116**, 21616–21622 (2019).
13. Brown, J. H. & Gillooly, J. F. Ecological food webs: high-quality data facilitate theoretical unification. *Proc. Natl. Acad. Sci. U. S. A.* **100**, 1467–1468 (2003).
14. Hatton, I. What Regulates Growth across Levels of Organization. *Unsolved Probl. Ecol.* 203 (2020).
15. Sheldon, R. W., Prakash, A. & Sutcliffe, W. H. The size distribution of particles in the ocean. *Limnol. Ocean.* **17**, 327–340 (1972).
16. Kerr, S. R. & Dickie, L. M. *The biomass spectrum: a predator-prey theory of aquatic production*. (Columbia University Press, 2001).
17. Polishchuk, L. V & Blanchard, J. L. Uniting discoveries of abundance-size distributions from soils and seas. *Trends Ecol. Evol.* **34**, 2–5 (2019).
18. Blanchard, J. L., Heneghan, R. F., Everett, J. D., Trebilco, R. & Richardson, A. J. From bacteria to whales: using functional size spectra to model marine ecosystems. *Trends Ecol. Evol.* **32**, 174–186 (2017).
19. Hatton, I. A., Heneghan, R. F., Bar-On, Y. M. & Galbraith, E. D. The global ocean size-spectrum from bacteria to whales. *Science Advances*, **7(46)**, p.eabh3732 (2021).
20. Trebilco, R., Baum, J. K., Salomon, A. K. & Dulvy, N. K. Ecosystem ecology: size-based constraints on the pyramids of life. *Trends Ecol. Evol.* **28**, 423–431 (2013).

Table S1 | Site information for the 141 food webs studied.

Ecosystem type	Food web	Location	Longitude	Latitude	Reference
Freshwater (stream)	Afon Berwyn	United Kingdom	-3.91	52.22	1,2
Freshwater (stream)	Afon Bidno	United Kingdom	-3.65	52.43	1,2
Freshwater (stream)	Afon Ceirw	United Kingdom	-3.83	52.84	1,2
Freshwater (stream)	Afon Dulais	United Kingdom	-3.90	52.04	1,2
Freshwater (stream)	Afon Gain	United Kingdom	-3.85	52.88	1,2
Freshwater (stream)	Afon Gwy 2012	United Kingdom	-3.73	52.45	1,2
Freshwater (stream)	Afon Hafren 2012	United Kingdom	-3.71	52.48	1,2
Freshwater (stream)	Afon Marteg	United Kingdom	-3.54	52.33	1,2
Freshwater (stream)	Allt a'Mharcaidh	United Kingdom	-3.85	57.12	1,2
Freshwater (stream)	Allt na Coire nan Con	United Kingdom	-5.61	56.76	1,2
Freshwater (stream)	G1 *	United Kingdom	-3.84	52.10	1,2
Freshwater (stream)	G2 *	United Kingdom	-3.85	52.10	1,2
Freshwater (stream)	L6 *	United Kingdom	-3.72	52.13	1,2
Freshwater (stream)	L7 *	United Kingdom	-3.73	52.13	1,2
Freshwater (stream)	Nant Clywedog Uchaf	United Kingdom	-3.98	52.15	1,2
Freshwater (stream)	Nant Gelli Gethin	United Kingdom	-3.41	52.65	1,2
Freshwater (stream)	Nant Pen y Cwm	United Kingdom	-3.41	52.64	1,2
Freshwater (stream)	Narrator Brook	United Kingdom	-4.02	50.50	1,2
Freshwater (stream)	River Bure	United Kingdom	1.20	52.82	1,2
Freshwater (stream)	River Duddon	United Kingdom	-3.16	54.41	1,2
Freshwater (stream)	River Etherow	United Kingdom	-1.83	53.49	1,2
Freshwater (stream)	River Fiddich	United Kingdom	-3.11	57.44	1,2
Freshwater (stream)	River Loddon	United Kingdom	-1.02	51.29	1,2
Freshwater (stream)	River Lyde	United Kingdom	-1.00	51.29	1,2
Freshwater (stream)	River Test	United Kingdom	-1.47	51.14	1,2
Freshwater (stream)	River Wensum	United Kingdom	0.95	52.78	1,2
Freshwater (stream)	Upper Duhonw	United Kingdom	-3.40	52.13	1,2

Freshwater (stream)	Upper Llugwy	United Kingdom	-3.92	53.11	1,2
Freshwater (stream)	Hardknott Gill	United Kingdom	-3.17	54.40	1,2
Freshwater (stream)	Mill Stream	United Kingdom	-2.18	50.68	1,2
Marine (rock pool)	AP2	Brasil (SP)	-45.41	-23.81	3,4
Marine (rock pool)	AP3	Brasil (SP)	-45.41	-23.81	3,4
Marine (rock pool)	AP4	Brasil (SP)	-45.41	-23.81	3,4
Marine (rock pool)	BP1	Brasil (SP)	-45.17	-23.52	3,4
Marine (rock pool)	CGP2	Brasil (SP)	-45.42	-23.83	3,4
Marine (rock pool)	CGP3	Brasil (SP)	-45.42	-23.83	3,4
Marine (rock pool)	CR1P1	Portugal	-9.49	38.71	3,4
Marine (rock pool)	CR1P2	Portugal	-9.49	38.71	3,4
Marine (rock pool)	CR1P3	Portugal	-9.49	38.71	3,4
Marine (rock pool)	CR1P4	Portugal	-9.49	38.71	3,4
Marine (rock pool)	CR2P1	Portugal	-9.49	38.71	3,4
Marine (rock pool)	CR2P2	Portugal	-9.49	38.71	3,4
Marine (rock pool)	CR2P3	Portugal	-9.49	38.71	3,4
Marine (rock pool)	CR2P4	Portugal	-9.49	38.71	3,4
Marine (rock pool)	F1P1	Mozambique	32.99	-25.97	3,4
Marine (rock pool)	F1P2	Mozambique	32.99	-25.97	3,4
Marine (rock pool)	F1P3	Mozambique	32.99	-25.97	3,4
Marine (rock pool)	F1P4	Mozambique	32.99	-25.97	3,4
Marine (rock pool)	F2P1	Mozambique	32.99	-25.97	3,4
Marine (rock pool)	F2P2	Mozambique	32.99	-25.97	3,4
Marine (rock pool)	F2P3	Mozambique	32.99	-25.97	3,4
Marine (rock pool)	F2P4	Mozambique	32.99	-25.97	3,4
Marine (rock pool)	FP1	Brasil (SP)	-45.16	-23.53	3,4
Marine (rock pool)	FXAP2	Brasil (CE)	-39.27	-3.22	3,4
Marine (rock pool)	FXBP2	Brasil (CE)	-39.27	-3.22	3,4
Marine (rock pool)	FXBP3	Brasil (CE)	-39.27	-3.22	3,4
Marine (rock pool)	GJAP4	Brasil (CE)	-39.23	-3.24	3,4
Marine (rock pool)	L1P1	Portugal	-9.34	39.29	3,4
Marine (rock pool)	L1P2	Portugal	-9.34	39.29	3,4
Marine (rock pool)	L1P3	Portugal	-9.34	39.29	3,4
Marine (rock pool)	L1P4	Portugal	-9.34	39.29	3,4
Marine (rock pool)	L2P1	Portugal	-9.34	39.29	3,4
Marine (rock pool)	L2P2	Portugal	-9.34	39.29	3,4
Marine (rock pool)	L2P3	Portugal	-9.34	39.29	3,4
Marine (rock pool)	L2P4	Portugal	-9.34	39.29	3,4
Marine (rock pool)	L3P1	Portugal	-9.34	39.24	3,4
Marine (rock pool)	L3P3	Portugal	-9.34	39.24	3,4
Marine (rock pool)	L3P4	Portugal	-9.34	39.24	3,4
Marine (rock pool)	L4P1	Portugal	-9.34	39.24	3,4
Marine (rock pool)	L4P2	Portugal	-9.34	39.24	3,4
Marine (rock pool)	L4P3	Portugal	-9.34	39.24	3,4

Marine (rock pool)	L4P4	Portugal	-9.34	39.24	3,4
Marine (rock pool)	PC1P1	Madeira	-16.83	32.78	3,4
Marine (rock pool)	PC1P2	Madeira	-16.83	32.78	3,4
Marine (rock pool)	PC1P3	Madeira	-16.83	32.78	3,4
Marine (rock pool)	PC2P2	Madeira	-16.83	32.78	3,4
Marine (rock pool)	PC2P3	Madeira	-16.83	32.78	3,4
Marine (rock pool)	PGSBP1	Brasil (SP)	-45.41	-23.82	3,4
Marine (rock pool)	PGSBP2	Brasil (SP)	-45.41	-23.82	3,4
Marine (rock pool)	PGUBP2	Brasil (SP)	-45.06	-23.47	3,4
Marine (rock pool)	PGUBP3	Brasil (SP)	-45.06	-23.47	3,4
Marine (rock pool)	PP1I1	Canada	-68.50	48.49	3,4
Marine (rock pool)	RMP1	Madeira	-16.82	32.65	3,4
Marine (rock pool)	RMP2	Madeira	-16.82	32.65	3,4
Marine (rock pool)	RMP3	Madeira	-16.82	32.65	3,4
Marine (rock pool)	RV1P1	Portugal	-9.48	38.70	3,4
Marine (rock pool)	RV1P2	Portugal	-9.48	38.70	3,4
Marine (rock pool)	RV1P3	Portugal	-9.48	38.70	3,4
Marine (rock pool)	RV1P4	Portugal	-9.47	38.70	3,4
Marine (rock pool)	RV2P1	Portugal	-9.47	38.70	3,4
Marine (rock pool)	RV2P2	Portugal	-9.47	38.70	3,4
Marine (rock pool)	RV2P3	Portugal	-9.47	38.70	3,4
Marine (rock pool)	RV2P4	Portugal	-9.47	38.70	3,4
Marine (rock pool)	SF1I3	Canada	-68.23	48.61	3,4
Marine (rock pool)	SP1	Brasil (SP)	-45.42	-23.83	3,4
Marine (rock pool)	WP3	United Kingdom	-4.08	50.32	3,4
Terrestrial (soil)	AEW01	Germany	9.33	48.48	5
Terrestrial (soil)	AEW02	Germany	9.35	48.38	5
Terrestrial (soil)	AEW04	Germany	9.24	48.40	5
Terrestrial (soil)	AEW05	Germany	9.41	48.42	5
Terrestrial (soil)	AEW07	Germany	9.26	48.40	5
Terrestrial (soil)	AEW08	Germany	9.38	48.38	5
Terrestrial (soil)	AEW09	Germany	9.42	48.37	5
Terrestrial (soil)	AEW17	Germany	9.24	48.40	5
Terrestrial (soil)	AEW18	Germany	9.23	48.37	5
Terrestrial (soil)	AEW25	Germany	9.42	48.48	5
Terrestrial (soil)	AEW27	Germany	9.47	48.40	5
Terrestrial (soil)	AEW30	Germany	9.37	48.37	5
Terrestrial (soil)	AEW49	Germany	9.48	48.45	5
Terrestrial (soil)	HEW01	Germany	10.32	51.19	5
Terrestrial (soil)	HEW02	Germany	10.37	51.21	5
Terrestrial (soil)	HEW03	Germany	10.31	51.27	5
Terrestrial (soil)	HEW04	Germany	10.53	51.37	5
Terrestrial (soil)	HEW05	Germany	10.24	51.26	5
Terrestrial (soil)	HEW06	Germany	10.24	51.27	5
Terrestrial (soil)	HEW10	Germany	10.46	51.09	5

Terrestrial (soil)	HEW11	Germany	10.40	51.10	5
Terrestrial (soil)	HEW12	Germany	10.46	51.10	5
Terrestrial (soil)	HEW13	Germany	10.31	51.24	5
Terrestrial (soil)	HEW16	Germany	10.37	51.18	5
Terrestrial (soil)	HEW17	Germany	10.23	51.28	5
Terrestrial (soil)	HEW21	Germany	10.32	51.19	5
Terrestrial (soil)	HEW22	Germany	10.36	51.34	5
Terrestrial (soil)	HEW36	Germany	10.41	51.11	5
Terrestrial (soil)	HEW47	Germany	10.38	51.18	5
Terrestrial (soil)	SEW01	Germany	13.85	52.90	5
Terrestrial (soil)	SEW02	Germany	13.78	52.95	5
Terrestrial (soil)	SEW03	Germany	13.64	52.92	5
Terrestrial (soil)	SEW04	Germany	13.85	52.92	5
Terrestrial (soil)	SEW05	Germany	13.89	53.06	5
Terrestrial (soil)	SEW06	Germany	13.84	52.91	5
Terrestrial (soil)	SEW07	Germany	13.69	53.11	5
Terrestrial (soil)	SEW08	Germany	13.93	53.19	5
Terrestrial (soil)	SEW09	Germany	13.81	53.04	5
Terrestrial (soil)	SEW18	Germany	13.92	52.86	5
Terrestrial (soil)	SEW35	Germany	13.85	52.91	5
Terrestrial (soil)	SEW36	Germany	13.75	52.95	5
Terrestrial (soil)	SEW37	Germany	13.78	52.94	5
Terrestrial (soil)	SEW41	Germany	13.91	52.91	5
Terrestrial (soil)	SEW43	Germany	13.93	52.90	5
Terrestrial (soil)	SEW48	Germany	13.84	53.05	5

[1] Perkins, D. M. *et al.* Bending the rules: exploitation of allochthonous resources by a top-predator modifies size-abundance scaling in stream food webs. *Ecol. Lett.* **21**, 1771–1780 (2018); [2] Perkins, D. M. *et al.* Data from: Systematic variation in food web body-size structure linked to external subsidies. (2021). doi:doi.org/10.6084/m9.figshare.9610112; [3] Mendonça, V. *et al.* What's in a tide pool? Just as much food web network complexity as in large open ecosystems. *PLoS One* **13**, e0200066 (2018); [4] Gauzens, B., Rall, B. C., Mendonça, V., Vinagre, C. & Brose, U. Biodiversity of intertidal food webs in response to warming across latitudes. *Nat. Clim. Chang.* **10**, 264–269 (2020); [5] Ehnes, R. B. *et al.* Lack of energetic equivalence in forest soil invertebrates. *Ecology* **95**, 527–537 (2014). * Fishless sites removed from prey body mass analysis (Methods; Fig. 5).

Table S2 | Summary output from the statistical analysis of (a) within-web and (b) across-web scaling relations. Slope values and 95% confidence intervals, along with the statistical significance of the relationships, were extracted from linear mixed-effects (within-web analysis) and ANCOVA (across-web analysis) models using the emmeans package in R.

	Relationship	Ecosystem type	Slope	Lower CI	Upper CI	SE	df	*t* ratio	*P* value
Within-web scaling	Predator biomass vs. prey biomass	Freshwater (streams)	0.61	0.50	0.71	0.06	165	10.98	<.0001
		Marine (rock pools)	0.74	0.66	0.82	0.04	100	17.87	<.0001
		Terrestrial (soils)	0.75	0.66	0.83	0.04	136	17.39	<.0001
		Overall	0.71	0.66	0.76	0.03	127	27.00	<.0001
	Predator biomass vs. PPmR	Freshwater (streams)	0.39	0.33	0.45	0.03	1950	12.74	<.0001
		Marine (rock pools)	0.79	0.71	0.87	0.04	1954	20.16	<.0001
		Terrestrial (soils)	0.30	0.21	0.38	0.04	1971	7.02	<.0001
	Predator biomass vs. omnivory	Freshwater (streams)	-0.21	-0.69	0.26	0.24	1439	-0.89	0.3743
		Marine (rock pools)	-0.17	-0.37	0.02	0.10	1981	-1.74	0.0815
		Terrestrial (soils)	1.60	1.37	1.83	0.12	1366	13.47	<.0001
Across-web scaling	Predator biomass vs. prey biomass	Freshwater (streams)	0.66	0.43	0.90	0.12	135	5.58	<.0001
		Marine (rock pools)	0.65	0.47	0.83	0.09	135	7.23	<.0001
		Terrestrial (soils)	0.67	0.26	1.09	0.21	135	3.18	0.0018
		Overall	0.66	0.52	0.79	0.07	137	9.74	<.0001
	Prey size vs. prey biomass	Freshwater (streams)	0.46	-0.26	1.19	0.37	131	1.27	0.2079
		Marine (rock pools)	0.24	-0.29	0.76	0.27	131	0.89	0.3777
		Terrestrial (soils)	0.43	-0.82	1.68	0.63	131	0.68	0.4991

Table S3 | Within-web analysis of predator-prey biomass scaling. Data were analysed with linear mixed-effects models. In Stage 1, random effects Models R1 to R3 were fitted using maximum likelihood (ML) and AIC tests were used to compare models. Results revealed that Model R1 (highlighted in bold), which included random variation in the power-law exponent (*k*) and constant (*c*) attributable to web identity, with a correlation term, best described the data. In Stage 2, Models F1 to F3 were fitted using ML and likelihood ratio tests were used to determine the significance of model parameters by comparing nested models. The *P* values of the likelihood ratio tests reveal that the more complex Model F1 was not a significantly better fit to the data than Model F2 ($P = 0.0894$**)** and that the simpler model, Model F3, was a significantly worse fit than model F2 ($P = <0.0001$**)** at $P = 0.05$ level. Thus, Model F2 (highlighted in bold), which includes ecosystem-type differences with respect to *c*, but not *k*, had the fixed-effect structure that best described the data, given the random-effects structure (Model R1). Parameters for Model F2 were then assessed by refitting the model using restricted maximum likelihood (REML).

Model	Df	AIC	logLik	R2 conditional	R2 marginal	Chisq	*P* value
Random-effects structure							
**R1. *k* * site | *c* * web identity**	**10**	**4735.29**					
R2. *k* * site + *c* * web identity	9	4736.79					
R3. *c* * web identity	8	4751.50					
Fixed-effects structure							
F1. *k* * ecosystem type | random = R1	10	4735.29	-2357.60	0.53	0.51		
**F2. *k* + ecosystem type | random = R1**	**8**	**4736.12**	**-2360.10**	**0.53**	**0.50**	**4.83**	**0.0894**
F3. *k* | random = R1	6	4826.35	-2407.20	0.51	0.42	94.23	< 0.0001

Table S4 | Within-web analysis of predator-prey biomass scaling with predator-prey size ratio and omnivory included. Predator omnivory and predator-prey body mass ratio (PPmR) were added as additional fixed effects to the final linear mixed-effects model characterising within-web scaling (Model F2; Table S2). Doing so reveals that the effects of omnivory and PPmR differ between ecosystem types, indicated by the significant interaction terms in the model. *P* values are estimated using Satterthwaite's method. Conditional $R^2 = 0.67$ and marginal $R^2 = 0.63$.

Fixed effect	Sum Sq	Mean Sq	NumDF	DenDF	F value	*P* value
Log10 (prey biomass)	204.25	204.25	1	148.74	493.003	< 2.2e-16
Ecosystem type	81.99	40.99	2	1150.40	98.95	< 2.2e-16
Log10 (PPmR)	213.77	213.77	1	1997.85	515.971	< 2.2e-16
Omnivory	7.67	7.67	1	1400.86	18.512	1.81E-05
Log10 (PPmR) : Ecosystem type	37.51	18.76	2	1989.78	45.27	< 2.2e-16
Omnivory : Ecosystem type	60.935	30.467	2	1467.83	73.54	< 2.2e-16

Table S5 | Sensitivity of scaling exponents to imposed trophic level cut-offs and vulnerability correction for (a) within-web and (b) across-web predator-prey biomass scaling. Cut-offs are based upon prey-averaged trophic level (TL) for a given predator: (i) all predators above or equal to a trophic level of 2.5 (presented in the main text and highlighted in grey) and (ii) all predators above or equal to a trophic level of 3. (iii) Exponent estimates are also presented for analyses where the vulnerability correction for prey was removed (see Methods). These results highlight that scaling exponents are largely robust to the choice of trophic cut-off and use of the vulnerability correction.

(a)

	**(i) TL >= 2.5**			**(ii) TL >= 3**			**(iii) TL >= 2.5 no vuln.**		
**Term**	**Exponent**	**Lower CI**	**Upper CI**	**Exponent**	**lower CI**	**Upper CI**	**Exponent**	**Lower CI**	**Upper CI**
Freshwater	0.61	0.50	0.71	0.66	0.54	0.77	0.62	0.48	0.76
Marine	0.74	0.66	0.82	0.74	0.64	0.85	0.69	0.59	0.80
Terrestrial	0.75	0.66	0.83	0.77	0.69	0.86	0.98	0.85	1.12
Across ecosystem types	0.71	0.66	0.76	0.74	0.68	0.79	0.76	0.68	0.83

(b)

**Term**	**Exponent**	**lower CI**	**Upper CI**	**Exponent**	**lower CI**	**Upper CI**	**Exponent**	**lower CI**	**Upper CI**
Freshwater	0.66	0.43	0.90	0.62	0.34	0.90	0.48	0.27	0.69
Marine	0.65	0.47	0.83	0.48	0.27	0.69	0.87	0.65	1.09
Terrestrial	0.67	0.26	1.09	0.66	0.23	1.09	0.83	0.28	1.37
Across ecosystem types	0.66	0.52	0.79	0.55	0.39	0.70	0.68	0.53	0.83

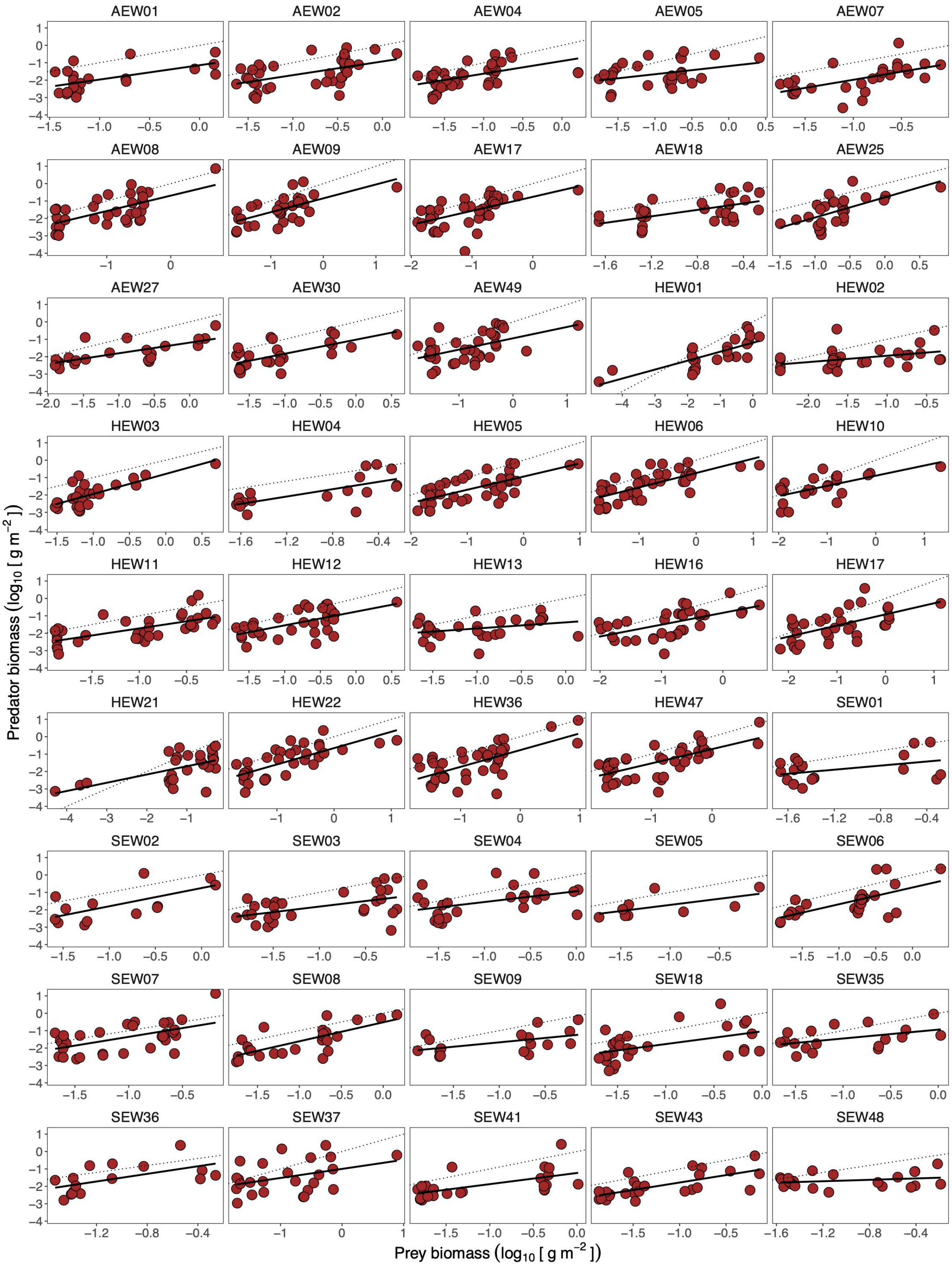

Figure S1 | Within-web biomass scaling in soil food webs. Each data point is a predator species and the summed biomass of its prey. The dashed line represents the 1:1 line.

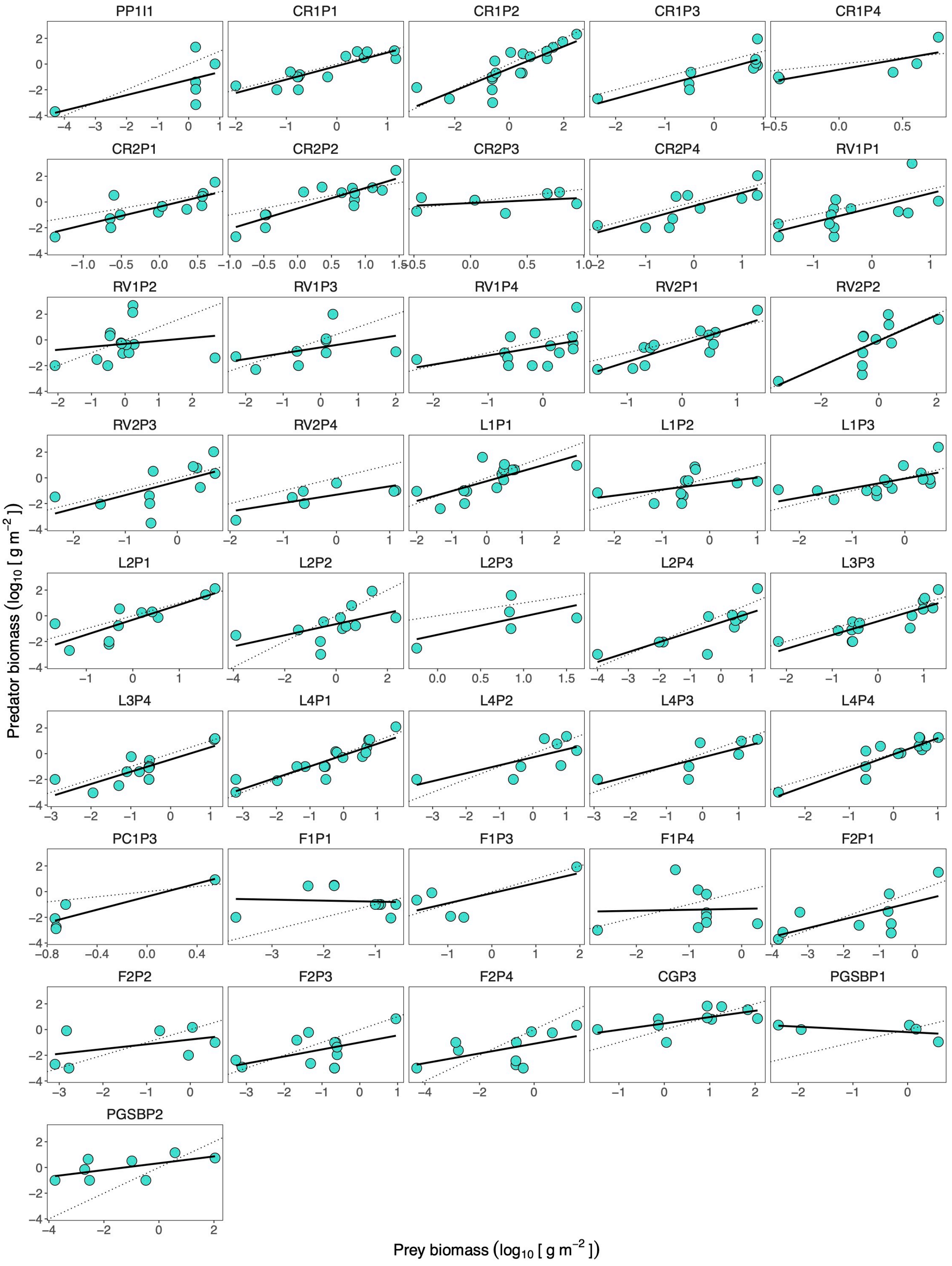

Figure S2 | Within-web biomass scaling in intertidal rock pool food webs. Each data point is a predator species and the summed biomass of its prey. The dashed line represents the 1:1 line.

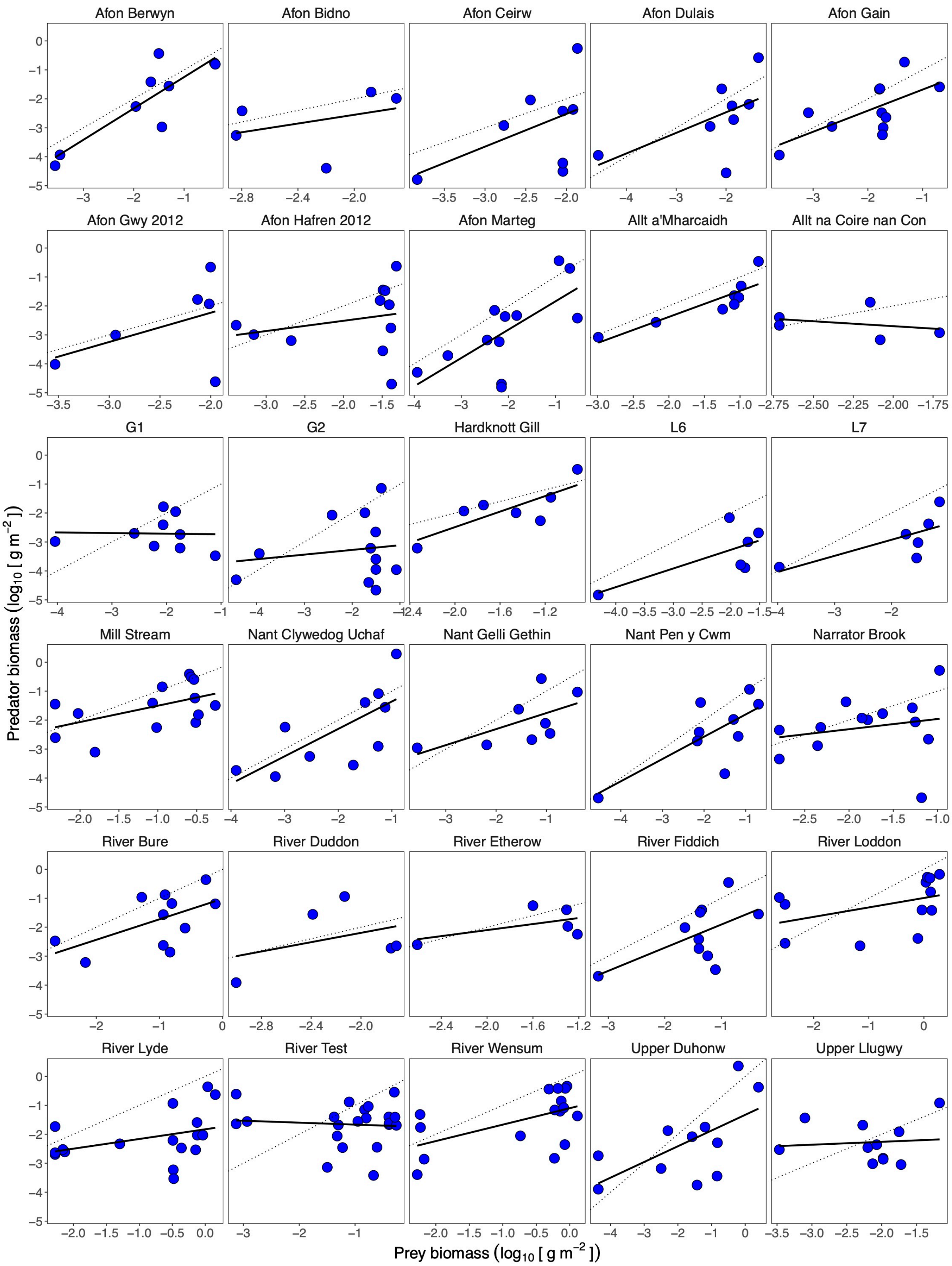

Figure S3 | Within-web biomass scaling in stream food webs. Each data point is a predator species and the summed biomass of its prey. The dashed line represents the 1:1 line.

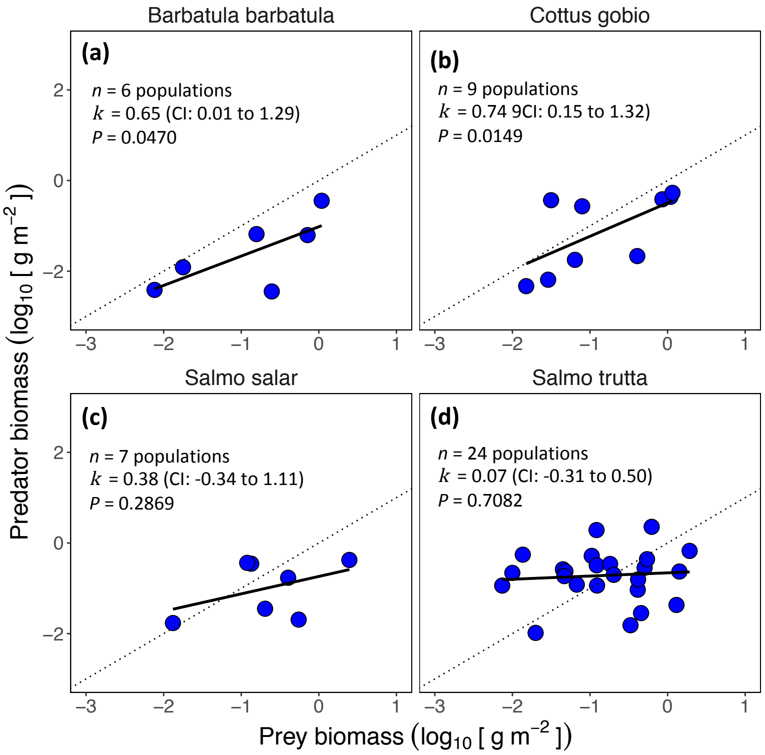

Figure S4 | Predator-prey biomass scaling for common fish species across stream food webs. Each regression is for a given predator species where each data point represents the biomass of that species and the summed biomass of its prey for an individual food web. The dashed line represents the 1:1 line. Whilst predator-prey scaling for (a) stone loach (*Barbatula barbatula*) and (b) bull head (*Cottus gobio*) display the characteristic near ¾-power scaling pattern, there is no evidence that salmonid biomass (c & d), and particularly the biomass of brown trout (*Salmo trutta*), is related to prey biomass, based upon *P* values of individual regression analyses.
