# Supplementary material for: Consistent predator-prey biomass scaling in complex food webs
Source: Nat Commun. 2022 Aug 25;13:4990. doi: 10.1038/s41467-022-32578-5 (PMC9411528; doi:10.1038/s41467-022-32578-5)
Supplement: Supplementary file 3 — Reporting Summary [file 41467_2022_32578_MOESM3_ESM.pdf]

nature portfolio

Corresponding author(s): Daniel Perkins & Ian Hatton
Last updated by author(s): 21/06/22

Reporting Summary

Nature Portfolio wishes to improve the reproducibility of the work that we publish. This form provides structure for consistency and transparency in reporting. For further information on Nature Portfolio policies, see our Editorial Policies and the Editorial Policy Checklist.

Statistics

For all statistical analyses, confirm that the following items are present in the figure legend, table legend, main text, or Methods section.

n/a	Confirmed	
☐	☒	The exact sample size (*n*) for each experimental group/condition, given as a discrete number and unit of measurement
☐	☒	A statement on whether measurements were taken from distinct samples or whether the same sample was measured repeatedly
☐	☒	The statistical test(s) used AND whether they are one- or two-sided<br>*Only common tests should be described solely by name; describe more complex techniques in the Methods section.*
☐	☒	A description of all covariates tested
☐	☒	A description of any assumptions or corrections, such as tests of normality and adjustment for multiple comparisons
☐	☒	A full description of the statistical parameters including central tendency (e.g. means) or other basic estimates (e.g. regression coefficient) AND variation (e.g. standard deviation) or associated estimates of uncertainty (e.g. confidence intervals)
☐	☒	For null hypothesis testing, the test statistic (e.g. *F*, *t*, *r*) with confidence intervals, effect sizes, degrees of freedom and *P* value noted<br>*Give P values as exact values whenever suitable.*
☒	☐	For Bayesian analysis, information on the choice of priors and Markov chain Monte Carlo settings
☐	☒	For hierarchical and complex designs, identification of the appropriate level for tests and full reporting of outcomes
☒	☐	Estimates of effect sizes (e.g. Cohen's *d*, Pearson's *r*), indicating how they were calculated

Our web collection on statistics for biologists contains articles on many of the points above.

Software and code

Policy information about availability of computer code

Data collection	No software was used for data collection
Data analysis	Data analysis was performed using the the R statistical platform (v. 3.4.1). Accompanying analysis R code is archived in a Figshare public repository (https://figshare.com/s/49f09c604b5be6df7838).

For manuscripts utilizing custom algorithms or software that are central to the research but not yet described in published literature, software must be made available to editors and reviewers. We strongly encourage code deposition in a community repository (e.g. GitHub). See the Nature Portfolio guidelines for submitting code & software for further information.

Data

Policy information about availability of data

All manuscripts must include a data availability statement. This statement should provide the following information, where applicable:

- Accession codes, unique identifiers, or web links for publicly available datasets
- A description of any restrictions on data availability
- For clinical datasets or third party data, please ensure that the statement adheres to our policy

Data was obtained from a global database of traits and food-web architecture (GATEWAy v.1.0; https://idata.idiv.de/ddm/Data/ShowData/283?version=3). The data used to generate Figures 2 & 4 are archived in a Figshare public repository (https://figshare.com/s/49f09c604b5be6df7838). Data included in the deposit are specifically designed for the replication of the analysis procedure. Therefore, researchers interested in using the data for purposes other than replicating our analyses are advised to obtain the raw data from the original sources.

Field-specific reporting

Please select the one below that is the best fit for your research. If you are not sure, read the appropriate sections before making your selection.

☐ Life sciences ☐ Behavioural & social sciences ☒ Ecological, evolutionary & environmental sciences

For a reference copy of the document with all sections, see nature.com/documents/nr-reporting-summary-flat.pdf

Ecological, evolutionary & environmental sciences study design

All studies must disclose on these points even when the disclosure is negative.

Study description

Meta-study of the biomass structure of freshwater (stream), marine (intertidal rock pool) and terrestrial (forest soil) food webs . Power-law relationships between predator and prey biomass were analysed and differences in power-law exponents between ecosystem types (freshwater, marine and terrestrial) tested.

Research sample

Available food web data was collated from a global database of traits and food-web architecture (GATEWAy v.1.0), where biomass and trophic interaction data were available across a large biomass gradient. Datasets for three ecosystem types met these requirements: 30 UK freshwater streams, a global compilation of 66 marine intertidal rock pools and 45 terrestrial soils of European forests.

Sampling strategy

Sample sizes were based upon established sampling protocols outlined in the original sources (Supplementary Table S1). With n = 2013 and n = 116, sample sizes were adequate for the analysis of within and across web scaling, respectively.

Data collection

Data was collated from a global database of traits and food-web architecture (GATEWAy v.1.0). Different methods were used for the collection of stream, intertidal rock pool and forest soil food webs and are given in the following sources:

Perkins, D. M. et al. Bending the rules: exploitation of allochthonous resources by a top-predator modifies size-abundance scaling in stream food webs. Ecol. Lett. 21, 1771–1780 (2018).

Perkins, D. M. et al. Data from: Systematic variation in food web body-size structure linked to external subsidies. (2021). doi:doi.org/10.6084/m9.figshare.9610112.

Mendonça, V. et al. What's in a tide pool? Just as much food web network complexity as in large open ecosystems. PLoS One 13, e0200066 (2018).

Gauzens, B., Rall, B. C., Mendonça, V., Vinagre, C. & Brose, U. Biodiversity of intertidal food webs in response to warming across latitudes. Nat. Clim. Chang. 10, 264–269 (2020).

Digel, C., Riede, J. O. & Brose, U. Body sizes, cumulative and allometric degree distributions across natural food webs. Oikos 120, 503–509 (2011).

Ehnes, R. B. et al. Lack of energetic equivalence in forest soil invertebrates. Ecology 95, 527–537 (2014)

Timing and spatial scale

The analysis is based upon data collected from single time points ("snapshots") in 141 survey sites. Further details on spatial scales and exact timing of data collection are provided in the following sources:

Perkins, D. M. et al. Bending the rules: exploitation of allochthonous resources by a top-predator modifies size-abundance scaling in stream food webs. Ecol. Lett. 21, 1771–1780 (2018).

Perkins, D. M. et al. Data from: Systematic variation in food web body-size structure linked to external subsidies. (2021). doi:doi.org/10.6084/m9.figshare.9610112.

Mendonça, V. et al. What's in a tide pool? Just as much food web network complexity as in large open ecosystems. PLoS One 13, e0200066 (2018).

Gauzens, B., Rall, B. C., Mendonça, V., Vinagre, C. & Brose, U. Biodiversity of intertidal food webs in response to warming across latitudes. Nat. Clim. Chang. 10, 264–269 (2020).

Digel, C., Riede, J. O. & Brose, U. Body sizes, cumulative and allometric degree distributions across natural food webs. Oikos 120, 503–509 (2011).

Ehnes, R. B. et al. Lack of energetic equivalence in forest soil invertebrates. Ecology 95, 527–537 (2014)

Data exclusions

Data were filtered to include only predators that had a prey averaged trophic level > 2.5. To ensure robust power-law fits to the data, we excluded from our analysis food webs that had fewer than five predators after this cut-off was applied, and where prey

	biomass varied by less than one order of magnitude. This resulted in 30 stream, 66 rock pool and 45 soil food webs for further analysis. Predators aggregated to coarse taxonomic groupings (e.g. zooplankton) were excluded from the within-web analysis shown in Fig. 2 (but included in the across-web analysis shown in Fig. 4). This resulted in the exclusion of 25 marine food webs (from the initial 66) for the within-web analysis, since the remaining number of consumers in the web was < 5.
Reproducibility	Reproducibility of the analysis is ensured through documented analysis code and data (see code and data availability statements)
Randomization	Randomization is not relevant to this study, which was not an experiment but concerns surveys of natural communities.
Blinding	Blinding is not relevant to this study, which was not an experiment but concerns surveys of natural communities.

Did the study involve field work? ☐ Yes ☒ No

Reporting for specific materials, systems and methods

We require information from authors about some types of materials, experimental systems and methods used in many studies. Here, indicate whether each material, system or method listed is relevant to your study. If you are not sure if a list item applies to your research, read the appropriate section before selecting a response.

Materials & experimental systems

n/a	Involved in the study
☒	☐ Antibodies
☒	☐ Eukaryotic cell lines
☒	☐ Palaeontology and archaeology
☒	☐ Animals and other organisms
☒	☐ Human research participants
☒	☐ Clinical data
☒	☐ Dual use research of concern

Methods

n/a	Involved in the study
☒	☐ ChIP-seq
☒	☐ Flow cytometry
☒	☐ MRI-based neuroimaging
